# Supplementary material for: Differentiation-independent activation of HPV genome replication by the lncRNA DINO
Source: mBio. 2026 Jul 29;17(9):e01443-26. doi: 10.1128/mbio.01443-26 (PMC13556205; doi:10.1128/mbio.01443-26)
Supplement: Supplementary file 1 [file mbio.01443-26-s0002.docx]

**Supplementary Figure 1. DINO expression increases during keratinocyte differentiation and DNA damage.**
**(A)** Endogenous DINO levels increase during differentiation induced by methylcellulose suspension. CIN612-9E cells were cultured under proliferative conditions or differentiated by suspension in 1.5% methylcellulose for 48 hours. Total RNA was extracted, and qRT-PCR was performed to measure endogenous DINO levels. Data from two independent experiments are presented as mean ± SEM. DINO levels increased significantly following suspension-induced differentiation, indicating that its upregulation is a feature of the differentiation process rather than a side effect of calcium-induced differentiation protocols. Statistical comparisons were performed using an unpaired t-test. **(B)** DINO expression increases in response to DNA damage in HPV-negative keratinocytes. Primary human foreskin keratinocytes (HFKs) were treated with 0.5 µg/mL doxorubicin for 24 hours, and DINO expression was measured by qRT-PCR. Doxorubicin treatment significantly increased DINO expression, demonstrating that DINO is induced by DNA damage independently of HPV infection. **(C)** DINO expression increases during calcium-induced differentiation of HPV-negative keratinocytes. HFKs were cultured under proliferative conditions or differentiated in KBM supplemented with 1.5 mM Ca²⁺ for four days. qRT-PCR was performed to measure the expression of the differentiation markers involucrin (IVL) and filaggrin (FLG), as well as DINO. Expression of all three genes significantly increased following differentiation, confirming that DINO upregulation is a general feature of keratinocyte differentiation.
